# Isolation Facilities for Highly Infectious Diseases in Europe – A Cross-Sectional Analysis in 16 Countries

**DOI:** 10.1371/journal.pone.0100401

**Published:** 2014-10-28

**Authors:** Stefan Schilling, Francesco Maria Fusco, Giuseppina De Iaco, Barbara Bannister, Helena C. Maltezou, Gail Carson, Rene Gottschalk, Hans-Reinhard Brodt, Philippe Brouqui, Vincenzo Puro, Giuseppe Ippolito, for the European Network for Highly Infectious Diseases project members

**Affiliations:** 1 Department for Internal Medicine, Staedtische Kliniken Moenchengladbach, Moenchengladbach, Germany; 2 Department for Infectious Diseases, National Institute for Infectious Diseases “Lazarro Spallanzani”, Rome, Italy; 3 Department for Internal Medicine, Azienda Ospedaliero, Universitaria Ospedali Riuniti delle Marche, Torrette, Italy; 4 Department for Infectious Diseases, The Royal Free Hospital, London, United Kingdom; 5 Department for Interventions in Health-Care Facilities, Hellenic Center for Disease Control and Prevention, Athens, Greece; 6 Department of Rare and Imported Pathogens, Health Protection Agency, Porton, United Kingdom; 7 Department for Infectious Diseases, Port Health Authorities, Frankfurt am Main, Germany; 8 Department for Infectious Diseases, Goethe University Frankfurt, Frankfurt am Main, Germany; 9 Department for Infectious Diseases and Tropical Medicine, Marseilles University, Marseille, France; University Medical Center Groningen, Netherlands

## Abstract

**Background:**

Highly Infectious Diseases (HIDs) are (i) easily transmissible form person to person; (ii) cause a life-threatening illness with no or few treatment options; and (iii) pose a threat for both personnel and the public. Hence, even suspected HID cases should be managed in specialised facilities minimizing infection risks but allowing state-of-the-art critical care. Consensus statements on the operational management of isolation facilities have been published recently. The study presented was set up to compare the operational management, resources, and technical equipment among European isolation facilities. Due to differences in geography, population density, and national response plans it was hypothesized that adherence to recommendations will vary.

**Methods and Findings:**

Until mid of 2010 the European Network for Highly Infectious Diseases conducted a cross-sectional analysis of isolation facilities in Europe, recruiting 48 isolation facilities in 16 countries. Three checklists were disseminated, assessing 44 items and 148 specific questions. The median feedback rate for specific questions was 97.9% (n = 47/48) (range: n = 7/48 (14.6%) to n = 48/48 (100%). Although all facilities enrolled were nominated specialised facilities' serving countries or regions, their design, equipment and personnel management varied. Eighteen facilities fulfilled the definition of a High Level Isolation Unit'. In contrast, 24 facilities could not operate independently from their co-located hospital, and five could not ensure access to equipment essential for infection control. Data presented are not representative for the EU in general, as only 16/27 (59.3%) of all Member States agreed to participate. Another limitation of this study is the time elapsed between data collection and publication; e.g. in Germany one additional facility opened in the meantime.

**Conclusion:**

There are disparities both within and between European countries regarding the design and equipment of isolation facilities. With regard to the International Health Regulations, terminology, capacities and equipment should be standardised.

## Introduction

The term highly infectious diseases (HID) defines mostly viral and bacterial infections that (i) are easily transmissible from person to person; (ii) cause a life-threatening clinical illness with no or few treatment options; and (iii) pose a threat for both health care workers and the public, thus requiring specific infection control measures and public health planning [1]. Included in this definition are viral haemorrhagic fevers (e.g. Filo-, Arena- or Bunyavirus infections); some respiratory syndromes (e.g. severe acute respiratory syndrome (SARS)-Coronavirus, or pneumonic plague); as well as any (re−) emerging infectious agent transmissible from person-to-person [2]. In Europe, HIDs have caused several events within the last decade: SARS affected eight European nations in 2003; Lassa virus has repeatedly been imported to Europe; and Crimean Congo Haemorrhagic Fever virus infections are increasing in several Mediterranean regions, and have also been imported to central Europe and the United Kingdom (UK) [3]–[7]. Recently detected agents like the new human Middle East Respiratory Syndrome Coronavirus underline the continuous challenge faced [8].

Patients with suspected or proven HIDs should be cared for in a clinical environment that provides safe, secure, high-quality, and appropriate care with optimal infection containment, prevention and control procedures [1]. Consensus statements on the operational management and design of isolation facilities have been published in Europe and the United States of America [1]; [9]. In addition, the European Commission has funded projects to enhance early recognition of cases by training front-line health care workers (HCWs) and standardizing diagnostic methodology [10]–[12]. Despite such efforts, no pooled data on isolation facilities resources, such as infrastructure design, technical equipment, capacity and access to intensive care, do exist.

The study presented was performed by the European Network for Highly Infectious Diseases, EuroNHID, and set up to compare the operational management, resources, and technical equipment among isolation facilities with recommendations published. Due to differences in geography, population density, national response plans, and experience with HIDs in participating countries, it was hypothesized that the level of adherence to recommendations may vary. The objective of this article is to present data about infrastructure design and resources, technical equipment, available personnel, and access to intensive care.

## Methods

### Setting

EuroNHID consists of infectious disease clinicians and public health officers with expertise in the management of HIDs identified via their National Health Authorities. Administrative and scientific aspects are managed by an Italian Coordination Team, and a Steering Committee, including partners from France, Germany, Greece, and the UK. Both committees started their work in 2007, and reported all organizational and scientific developments to the project members. Names and countries of all project members are listed above.

### Study design

Three checklists were developed and based on project-member’s experience, available literature, national preparedness plans, and guidelines of international authorities for the management of HIDs [13]. Each checklist contained questions addressing specific items of interest, and answers were structured as open-ended, closed, semi-open or free text options. Overall, the checklists included 44 items and 148 specific questions.

Checklist one contained three major sections: Infrastructure; technical equipment; and personnel management. A fourth section on Emergency Departments was not obligatory [14]. All checklists can be downloaded after free registration from: www.EUNID.eu.

The checklists underwent a pilot application to identify structural gaps and sources of misinterpretation [13]. The facilities of the Steering Committee members and one external facility applied the checklists and cross-checked all itmes for their applicability, but no external validation process was conducted. Following minor changes, the checklists were disseminated to all eligible facilities until December 2009 and expected to be re-submitted within three months. Data provided were cross-checked by personal on-site visits by a coordination team member in all but four centres enrolled. Investigator (the National Project Representative) and observer (the Coordination Team member) bias may be ruled out, as all data recorded were cross-checked on-site and agreed with the facilities operational manager or director.

### Participants

Until November 2009, the Coordination Team collected data on eligible isolation facilities to be included into the survey. National Health Authorities of all European Union (EU) member states were contacted to identify their will to participate. Participating National Health Authorities were requested to identify clinical facilities responsible for the admission, assessment, and care of HID patients in their country, and to nominate a National Project Representative.

### Role of the funding source

EuroNHID was co-funded by the European Commission/Director General for Consumers and Health (DG SANCO) for an initial period of 36 months and extended to 42 months without additional funding. A technical and scientific report on all data collected during the project was delivered to the Commission in December 2010. For details please follow: http://ec.europa.eu/research/health/infectious-diseases/emerging-epidemics/projects/143_en.html.

## Results

### Participants

Until mid of 2010, 48 isolation facilities in 16 European countries were recruited into this survey (Table 1). Recruitment was closed by the end of 2009 with the exception of Norway, who received questionnaires by end of 2009 and submitted data by spring of 2010. Data collection and on-site visits were finalised in spring 2010. Except for Norway, all participating countries were EU member states. Data were collected from all existing facilities in participating countries except for Spain, where data were only collected in the Catalonia region. Overall, the median feedback rate for checklist 1 was 97.9% (n = 47/48), with a range from n = 7/48 (14.6%) to n = 48/48 (100%) of centres providing valid answers to specific questions. All data provided in this article represent a minimum feedback from 44 facilities (>90%).

**Table 1:** Number of isolation facilities, existence of High Level Isolation Units, and isolation beds for highly infectious patients per participating country.

Participating country	Overall number offacilities enrolled^#^	Overall number ofisolation beds	Number of isolation beds/millionof population*
**Austria**	1	24	2.87
**Bulgaria**	2	64	8.46
**Denmark**	1	56	10.12
**Finland**	2 (1)	57	10.65
**France**	12 (5)	112	1.73
**Germany**	8 (6)	44	0.54
**Greece**	6 (1)	20	1.77
**Ireland**	2 (1)	4	0.89
**Italy**	2 (2)	5	0.08
**Luxembourg**	1	15	29.87
**Malta**	1	3	7.26
**Norway**	1 (1)	4	0.82
**Poland**	1	2	0.05
**Slovenia**	1	2	0.97
**Spain**	5	38	0.58^§^
**United Kingdom**	2 (1)	3	0.05

(#): Numbers in brackets indicate the amount of High Level Isolation Units per country, if existing. (*): Total population calculated on the basis of Eurostat [30]. (§): Facilities enrolled are located in the Catalonia region, only.

### Background data on facilities evaluated

The majority of facilities were constructed or underwent major re-construction in the year 2001 or later (n = 32/47; 66.7%) and are located on the same campus or in direct connection with a general hospital (n = 41/48; 85.4%). Most facilities have designated rooms (n = 23/48; 47.9%) or wards (n = 18/48; 37.5%) located within other hospital structures, and seven facilities are located within a separate building (n = 7/48; 14.6%). National guidelines for the construction of isolation facilities were available for 17 facilities whereas 29 were (re−) constructed in the absence of specific requirements (37% and 63%, respectively). Three facilities accept paediatric (6.3%), and 14 (29.2%) accept adult patients, only, whereas most facilities provide care for both (n = 31/48; 64.6%). The number of isolation beds per facility ranges from one to 56, with a median of 9.3 beds for adult and 7.2 for paediatric patients. With respect to the overall population, the number of beds ranges from 0.05 to 17.9 per one million inhabitants (median: 1.7) (Table 1). The majority of all facilities uses their beds on a daily basis for routine patients (n = 37/48; 77,1%), while the remainder provide bed capacities reserved for HID cases, only (n = 11/48, 22.9%).

Except for the United Kingdom, all countries (n = 46/48; 95.4%) use barrier nursing techniques with high level Personal Protective Equipment (hl-PPE) (21). 37 facilities (n = 37/46; 80.4%) have a response time of less than five hours after a case is notified, the remainder demand up to 10 hours to be fully operational (n = 9/46; 19.6%). Only eight (16.7%) of all facilities evaluated lacked any experience in the management of suspected or proven HID cases. Among the remainder, most had experience with infections due to SARS (n = 13/48; 27.8%), and viral haemorrhagic fevers (n = 9/48; 18.8%).

### Technical equipment

**Figure 1:**
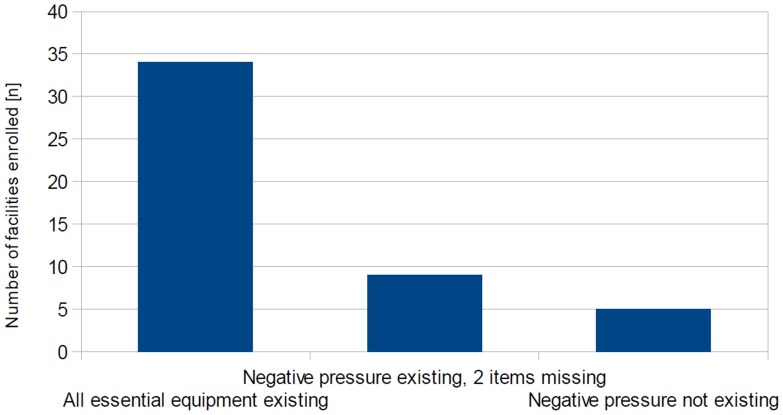
Existence of technical equipment essential for infection control and HCW’s safety. Number of enrolled facilities and adherence to recommended equipment (overall response n = 48/48). Access to all essential equipment was reported from 34 facilities; negative pressure existing, but 2 other items missing in 9 facilities; and no negative pressure available in 5 facilities.

Essential technical requirements for isolation facilities includes: Negative pressure in the isolation room(s); an anteroom; aerosol-tight doors and windows; High Efficacy Particulate Air (HEPA) filtration of exhausted air; and a surface of walls, floors and ceiling withstanding disinfection procedures [1]. All but five facilities enrolled (n = 43/48; 89.6%) provide negative pressure in the isolation area, and the majority (n = 34/48; 70.8%) is also equipped with all other essential equipment investigated. In facilities with two or less additional items, most often HEPA filtration of exhausted air and anterooms were missing (n = 7 and 5/14 facilities, respectively) (Figure 1).

Additional technical requirements not considered essential, but benefitting infection control and HCW safety, includes: self-closing doors; audio and/or visual negative pressure indicators; an exclusive evacuation pathway; and an internal communication system (for HCW-HCW and HCW-patient communication) [1]. At least 3 additional technical requirements are found in the majority of facilities (n = 34/48; 70.8%). Most often, an internal communication system (n = 43/48; 89.6%) and negative pressure indicators (n = 40/48; 83.3%) were present, while an exclusive evacuation pathway was missing in half of all facilities enrolled (n = 27/48, 56.3%).

### Access to Intensive Care

**Figure 2:**
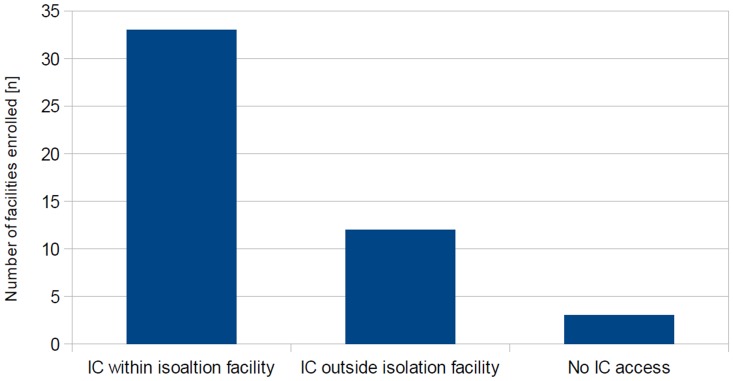
Access to and location of Intensive Care beds for HID patients. Number of facilities enrolled with Intensive Care (IC) capacity and location of IC beds (overall response n = 48/48). 33 facilities provide IC beds within the isolation area, and 12 in designated rooms on standard IC wards.

Thirty-three facilities (68.8%) provide intensive care, available for patients within the isolation facility. Twelve facilities (25%) rely on a designated isolation room within standard intensive care units, three (6.3%) have no access to intensive care capacities (Figure 2). Equipment for the monitoring of vital parameters and advanced life support is available in all 48 facilities. In contrast, mechanical ventilators are available either permanently in twelve (25%) or on call in thirty-two facilities (66.7%), but not accessible at all in four (8.3%). Forty-two facilities (87.5%) have either permanent or on-call access to blood gas analyzers, whereas six have not (12.5%). Hence, out of forty-five facilities reporting intensive care provision, one (2.2%) lacks a mechanical ventilator, and three (6.7%) have no ability to monitor their ventilation therapy.

### Personnel

**Figure 3:**
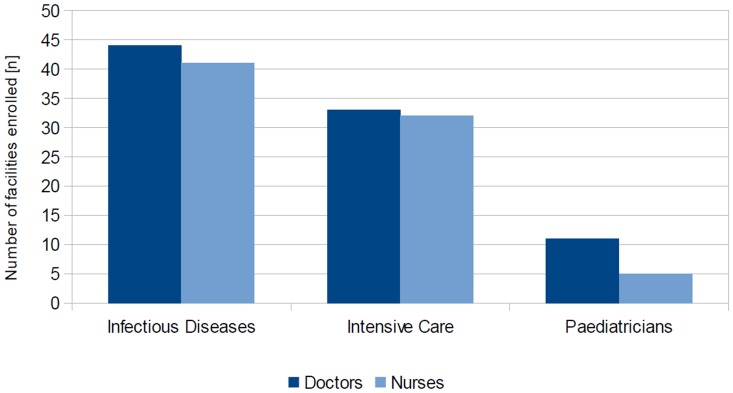
Access to specifically trained personnel. Number of facilities enrolled with access to specifically trained personnel (overall response n = 48/48). Access to Infectious Disease and Intensive Care doctors or nurses is provided in the majority of facilities, but specifically trained personnel for paediatric patients is rarely available.

Almost all facilities (93.6%; n = 44/47) report permanent access to specifically trained infectious diseases doctors. 45/48 (93.75%) facilities report providing intensive care, but only 34 of those (75.5%) have access to specifically trained intensive care specialists. In addition, out of 34 facilities indicating to provide care for pediatric HID patients, only 11 (32.4%) report either permanent or on-call access to pediatricians. Compared with available doctors, even fewer facilities report access to specifically trained infectious diseases nurses (n = 41/48; 85.4%), but intensive care nurses are available in a comparable number of facilities (n = 32/45; 71.1%) (Figure 3).

All but two facilities indicate 24-hour access to specifically trained medical and non-medical staff (n = 45/47; 95.7%), although 72.9% (n = 35/48) only have specific protocols to contact staff responsible for the operation of the facility. A shift plan to limit the number of HCWs exposed to patients as well as the duration of working under hl-PPE exists in 70.8% of facilities (n = 34/48). Permanent access to technicians was lacking in eleven facilities (n = 11/48; 22.9%).

## Conclusions

All participating facilities were evaluated for their adherence to recommendations for the operational management of isolation facilities published [1]. As hypothesized, the level of adherence to recommendations varied, both within and between participating countries.

Hence, three categories of isolation facilities representing different levels of adherence were defined:

(i) High Level Isolation Units (HLIU), defined as operating independently from other hospital resources and specifically equipped; (ii) Isolation Rooms, defined as specifically equipped but only partially independent from other hospital resources; and (iii) Referral Centers, neither specifically equipped nor functionally independent.

**Figure 4:**
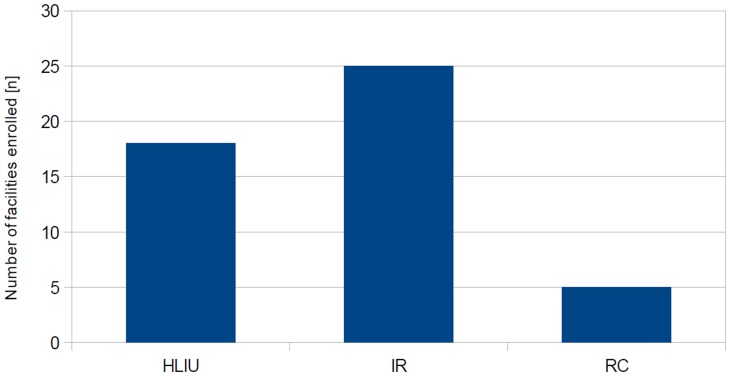
Classification of enrolled facilities by adherence to EUNID recommendations. Number of facilities classified by adherence to published recommendations: 18/48 facilities fulfilled the criteria of ‘High Level Isolation Units’ (HLIU); 25 met criteria for ‘Isolation Rooms’ and 5 for ‘Referral Centres’.

According to this classification, all facilities enrolled were categorized as shown in Figure 4. Few facilities completely fulfilled recommendations for HLIUs (n = 18/48; 37.5%). Six of those are located in Germany, five in France, two in Italy, and one each in the UK, Finland, Greece, Ireland, and Norway. Such HLIUs are constructed and equipped for the admission, assessment and long-term critical care of HID patients.

Most facilities assessed did partially meet the recommendations, but lacked infrastructural components, or preconditions for personnel management (n = 25/48; 52%). Such were labeled ‘Isolation Rooms’ considered effective to assess HID patients and provide short-term medical care until a patient can be transferred to a HLIU. Of these, five each are located in France, Greece, and Spain; two in Germany; and one each in Denmark, Finland, Ireland, Luxembourg, Malta, Poland and Slovenia.

The remaining facilities (n = 5/48; 10.4%) are considered to function as ‘Referral Centers’ where suspected HID cases can be assessed within daily routine, but even short-term critical care cannot be provided without an increased risk of nosocomial infections. Two of such facilities are located in Bulgaria and France, each, and one in Austria.

## Discussion

Within the last decades, autochthonous outbreaks or imported cases of HIDs have affected Europe with significant impact, and new pathogens are emerging. Most European countries have established national response plans including specialised clinical care facilities for the management of such scenarios [3]–[7], [15]–[17]. This article presents the first standardised analysis on the operational management, infrastructure, and technical equipment of 48 isolation facilities in 16 European countries. Data provided may support national authorities to assess their level of preparedness but have to be adapted to single member states’ needs and resources. With regard to the implementation of the International Health Regulations [18], terminology, capacities and equipment accessible in isolation facility should be documented and standardised on a European level.

Although all EU member states were invited to join the project by DGSANCO, only 16 agreed to participate, thus data presented are not representative for the EU in general. A major limitation of this study is the time elapsed between data collection and publication: e.g. in Germany one another facility has opened in the meantime. Hence, a periodical re-assessment of facilities and countries should be achieved, aiming for a permanently updated record of national preparedness. In addition, the checklists applied did not undergo an external validation process (e.g. a Delphi Cycle) due to limitations of funds and project duration, and present published guidelines, only. Hence, within the data collection process, misinterpretations occurred and items needed to be excluded from the data analysis presented here.

Half of all countries assessed have at least one HLIU according to the recommendations [1]. Compared to the definition, 18 facilities fulfilled the criteria applied, but the majority of all facilities enrolled could not operate independently from other facilities. The high rate of incompliance with the recommendations depicted here can be explained by the absence of legally binding documents by the time most facilities were constructed. After SARS and the US anthrax attacks in 2001, the political will was to enhance public preparedness, but no standard was agreed on. Only few countries (e.g. the UK) had specific guidelines at hand, but the vast majority of facilities were constructed using laboratory- or operational theatre guidelines adapted to local needs and infrastructure prerequisites. An earlier inventory of isolation rooms in Europe by our group faced this problem, when EU countries were asked to report on negative pressure rooms for HID patients [19]: The overall number of rooms exceeded most expectations, but both technical and infrastructural conditions were not well defined. Hence, an overreporting of any isolation room' with available led to the impression of a well prepared health system, although other aspects of equipment and personnel were not addressed. Given the definitions for different levels of isolation facilities proposed now, a reassessment of the EU's capacities could lead to more accurate data. Furthermore, the allocation of funds for hospital's biological event preparedness has decreased since the SARS pandemic, and is not harmonised within the EU. Hence, as our data show, the level of adherence to the recommendations mirrors the economical situation in the EU, with the highest level of adherence in the economical most stable countries. The situation in the United States of America is comparable: a consensus on the operational management of isolation units consistent with the European approach exists, but only three facilities fulfill those criteria, although hospital preparedness for biological events is mandatory [9], [20]. The contrary can be found in Israel, where the capability to manage HID patients is mandatory for every public hospital, and dedicated HLIUs are absent [21].

As HLIUs are designed for single imported cases or small clusters in the beginning of an outbreak, only, they do not compensate for surge capacity planning in major biological events [1]. Besides infrastructure and equipment, additional features which support effective functioning of such specialised centres were addressed in this survey and recently published [22]–[25]. Access to a transportation system for HID patients plays an important role in distribution of isolation facilities and is reflected by the overall amount isolation facilities per country. Balancing the necessary access to a focus of expertise with available transport logistics leads to either a centralised or a de-centralised approach to the location of specific facilities, and ambulance dispatch recording can be of use in early warning systems for outbreak detection [26].

Most documented HID cases demanded supportive intensive care [16], [17], [27], and access to such is considered essential for HLIUs [1]. In order to allocate funds most effectively, a balance between infection control excellence and operational feasibility should be sought. Basic specifications for HLIU provision, including access to intensive care equipment and trained personnel, should be developed in order to facilitate a common standard of best clinical practice. Key issues are the composition of a permanently available multidisciplinary medical and non-medical team, the ability and physical fitness to work with PPE, and reliable timelines and shifts to prevent accidents or mistakes due to exhaustion [1], [28]. Data depicted in this article do highlight available personnel as the most crucial pitfall in operating isolation facilities: Compared to the complicane with technical equipment recommendations, the lack of specifically trained staff is surprising, especially for HCWs trained in intensive and paediatric care. It should be mentioned that all facilities with experience in proven HIDs provide more sophisticated human resources management such as shift-and surge capacity planning as an outcome of their experience.

The overall investment for an isolation facility, and HLIUs in particular, is high, in terms of costs for construction and maintenance, medical and infection control equipment, human resources and training activities. Even in time of economical constraints, employers “are obliged to ensure the health and safety of workers”, as defined by the European Commission [29]. Without adequate protection, HCWs may acquire and transmit infections, with potential dramatic impact on the health system, and the economic solidity of a country, as documented during the SARS epidemic in Canada. For these reasons, we believe that specialized isolation facilities, and HLIUs in particular, still represent a critical infrastructure to which each EU Member State should have access, within the country or through pre-defined agreements with neighbouring countries where such facilities are available. Whether such capacities should be reserved for HID cases, only, or also used on routine basis, is part of an ongoing discussion. Financial constraints within hospitals and the host country may benefit from a routine-use concept, although such facilities demand more frequent maintenence due to damage on technical equipment. In addition, access to routine use beds for training of HCWs is reduced, and patients need to be relocated to other rooms once the facility receives an HID case. In contrast, reserved bed capacities demand additional personnel and material for routine functional checks, thus considered cost- ineffective. The major benefit from reserved capacities is a full access for training and no delay in patients admission since no evacuation of others is needed.

The WHO IHRs define the need of preparedness for infectious diseases outbreaks, but a European, if not international, consensus on the funding and minimum number of isolation capacities is not in sight. Although HIDs are rare events, they cause dynamic and often rapidly evolving issues in need of comprehensive solutions and may challenge the capacity of healthcare systems. Leadership and funding at both national and European level are required to harmonize preparedness plans, terminology and communication to weaken the impact of future infectious disease outbreaks with cross-border potential. In order to achieve a balance between saving lives and protecting HCWs in hazardous environments, national and international collaboration should continue to share experiences, and provide standardized training and equipment.
